# Supplementary material for: Impacts of Radio-Frequency Electromagnetic Field (RF-EMF) on Lettuce (Lactuca sativa)—Evidence for RF-EMF Interference with Plant Stress Responses
Source: Plants (Basel). 2023 Feb 28;12(5):1082. doi: 10.3390/plants12051082 (PMC10005510; doi:10.3390/plants12051082)

SUPPLEMENTARY MATERIALS

Impacts of radio-frequency electromagnetic field (RF-EMF) on lettuce (*Lactuca sativa*)– Evidence for RF-EMF interference with plant stress responses

Tran Nam Trung [a,*], Luca Jokic [a], Julian Keller [b], Jens Uwe Geier [b] and Ralf Kaldenhoff [a]

[a] Applied Plant Sciences, Department of Biology, Technical University Darmstadt, Darmstadt, Germany

[b] Forschungsring e.V., Darmstadt, Germany

* Corresponding author (E-mail: *tran@bio.tu-darmstadt.de*)

CONTENTS

Calculation of the Photochemical Stress Index (PSI) ………………………………………………… 2

Time course of 8 OJIP parameters F_V/F_M, F_V/F_O, ΨE_O, δR_O, dVG/dt_O, RC/ABS, RC/CS_O and Area ……………………………………………….. 4

Field trial's meteorological data ……………..…………………………………………………. 25

CALCULATION OF THE PHOTOCHEMICAL STRESS INDEX (PSI)

Based on the integrated biomarker response (IBR) methodology, we calculated the photochemical stress index (PSI) **[30, 31, 32]**. The PSI is an integrated indicator that reflects the overall photosynthetic efficiency and is computed from 19 separate OJIP variables that correspond to different biochemical processes of photosynthesis **[32]**. From each measurement, a single PSI value is computed.

Figure SM1 depicts the 19 OJIP variables. 15/19 parameters are derived directly from the OJIP curve using PEA Plus software (Hansatech) in F1-Mode, and P_G was calculated according to Panda et al. 2005 **[51]**.

$$P_G = \frac{(W_{E,\ 100\ \mu s} - W_{100\ \mu s})}{W_{100\ \mu s}\,(1 - \ W_{E,\ 100\ \mu s}\, V_J)}\,\frac{F_0}{F_m - F_0}$$

Where

$$W_{E,100\ \mu s} = 1 - \left(1 - W_{300\ \mu s}\right)^{1/5}$$

$$W_{100\ \mu s} = \frac{F_{100\ \mu s} - F_{50\ \mu s}}{F_{2\ ms} - F_{50\ \mu s}}$$

$$W_{300\ \mu s} = \frac{F_{300\ \mu s} - F_{50\ \mu s}}{F_{2\ ms} - F_{50\ \mu s}}$$

The other three variables were determined with the following equations:

$$\varphi_{Do} = 1 - \varphi_{Po}$$

$$TR_0/DI_0 = \frac{\mathrm{TRo/RC}}{\mathrm{DIo/RC}}$$

$$RC/CS_0 = \frac{\mathrm{ABS/CSo}}{\mathrm{ABS/RC}}$$

To calculate PSI, the first step was to normalize each OJIP variable (X) using the general mean (m) and standard deviation (s) of all measurements taken at the same time point ((treated and control plants combined) to produce the Y-scores:

$$Y = \frac{\mathrm{X} - m}{s}$$

Z-scores are calculated using Y-scores. Z = Y if the associated OJIP parameter is positively correlated with photosynthesis. Z = -Y if the associated OJIP parameter is negatively correlated with photosynthesis. In our cases, φ_{Do}, W_k, PG and DI/CS_o all have a negative correlation with photosynthesis; higher values of these parameters indicate a decrease in photosynthetic activity.

From the Z-scores, S-scores were calculated as:

$$S = Z - Z_{min}$$

Z_{min} was the smallest value for all Z-scores across all measurements (treated and control plants combined), and it was always negative. All S-scores were larger than or equal to zero.

Finally, the 19 S-scores, which correspond to 19 OJIP parameters, were plotted on a spider plot, as shown in **Figure SM1.** PSI was computed as the total plot area using the following equations:

$$PSI = \sum_i A_i$$

With A_i being the area between two consecutive S-scores S_i and S_{i+1}:

$$A_i = \frac{S_i}{2} sin\, \beta\, (S_i\, cos\, \beta + S_{i+1}\, sin\, \beta)$$

$$\beta = \tan^{-1} \frac{S_{i+1} \sin \alpha}{S_i - S_{i+1} \cos \alpha}$$

$$\alpha = \frac{2\pi}{19}$$

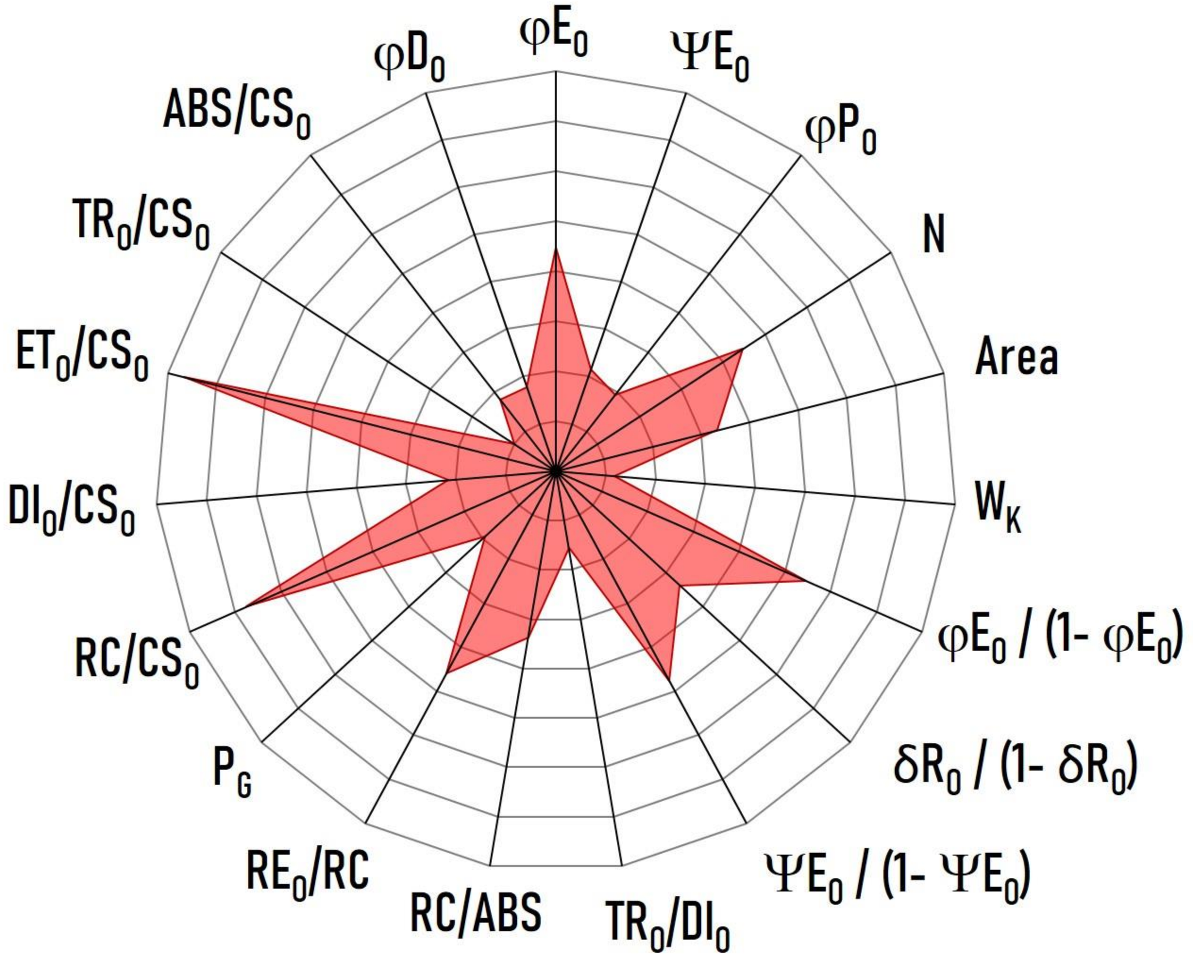

Figure S1: Computation of the photochemical stress index (PSI). 19 S-scores, which correspond to 19 OJIP parameters, are plotted on a spider plot. PSI was computed as the total plot area (red).

TIME COURSE OF 8 OJIP PARAMETERS F_V/F_M, F_V/F_O, ΨE_O, δR_O, dVG/dt_O, RC/ABS, RC/CS_O and AREA

In the following diagrams, we show the time course of the following 8 OJIP parameters during all 8 indoor and outdoor experiments: F_V/F_M - the maximal quantum yield of PSII photochemistry; F_V/F_O - the maximal quantum yield of oxygen-evolving complex (OEC); ΨE_O - quantum yield of the electron transport in the intersystem electron chain (from Q_A to plastocyanin PC); δR_O - quantum yield of the reduction of end acceptors at PSI side; dVG/dt_O - excitation energy transfer between the reaction centers; RC/ABS - effective antenna size; RC/CS_O - reaction center density; Area - pool size of reduced plastoquinone (PG) on the reducing side of PS II **[34 35, 36]**. Yellow: RF-EMF exposed plants; white:

control plants. Statistical symbols: * - statistically significant (0.01 < p < 0.05); ** - statistically highly significant (p < 0.01).

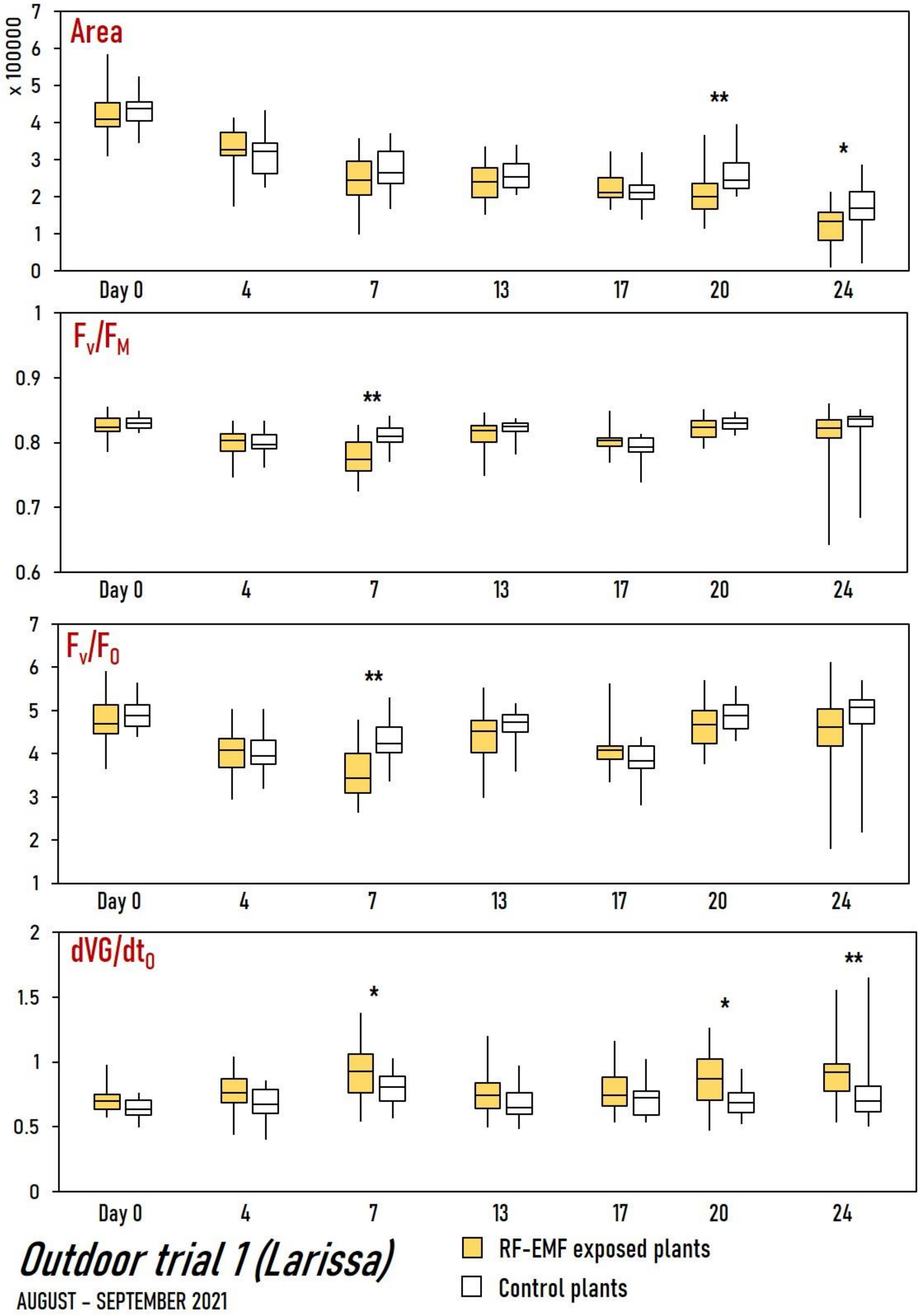

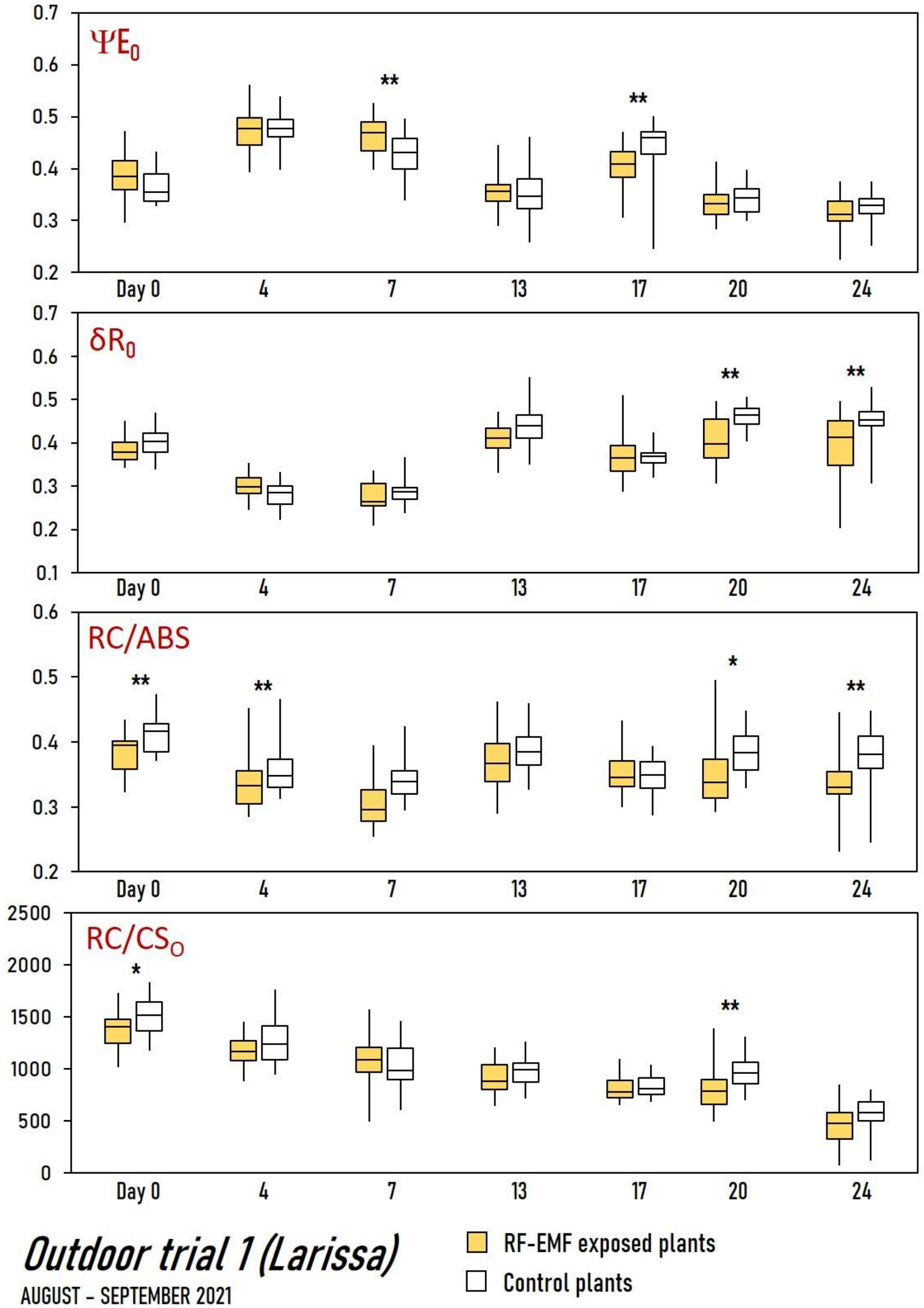

Outdoor trial 1 (Larissa)

AUGUST – SEPTEMBER 2021

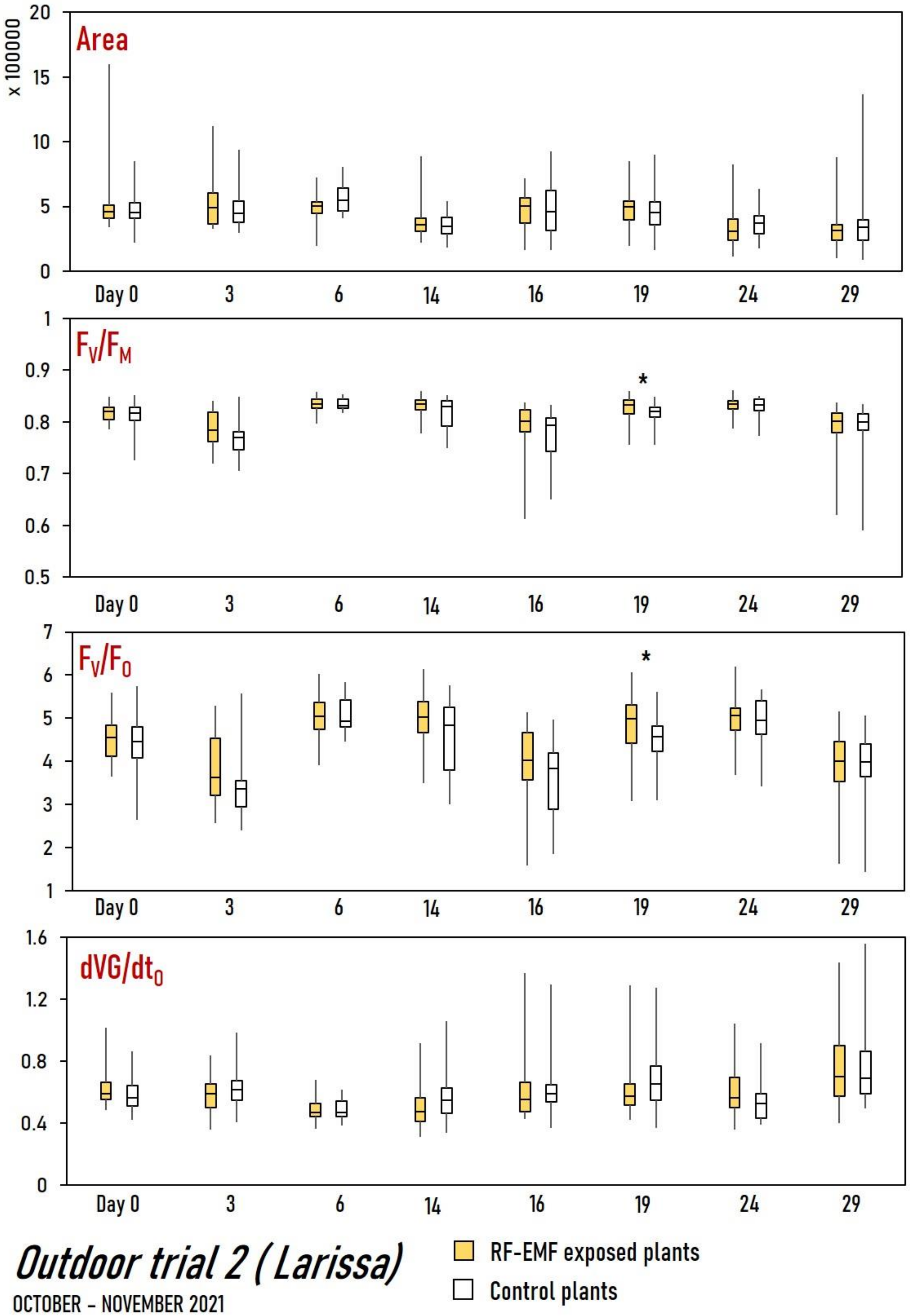

Outdoor trial 2 (Larissa)

OCTOBER – NOVEMBER 2021

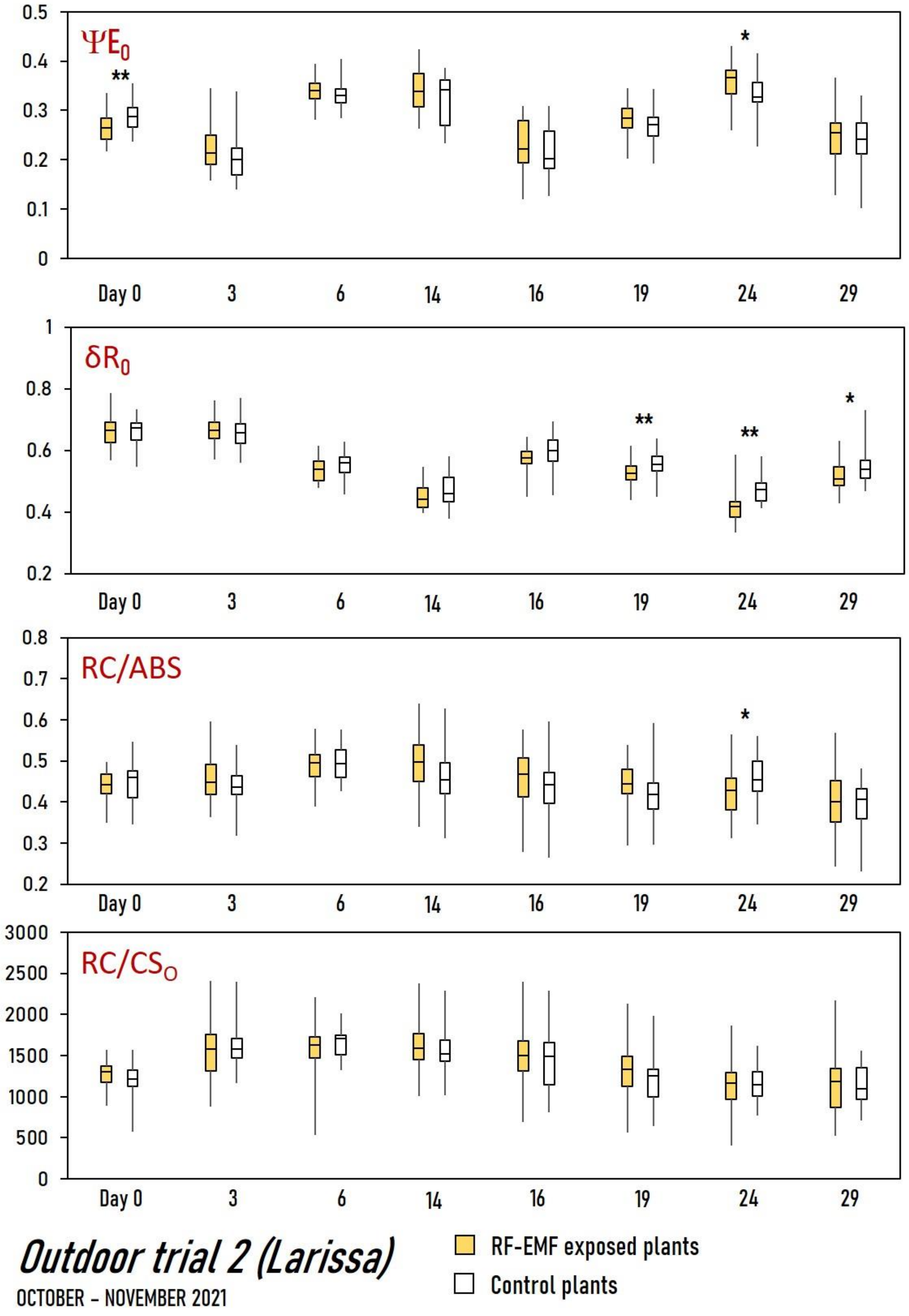
ΨE0
δR0
RC/ABS
RC/CSO
Day 0
3
6
14
16
19
24
29
RF-EMF exposed plants
Control plants
Outdoor trial 2 (Larissa)
OCTOBER – NOVEMBER 2021

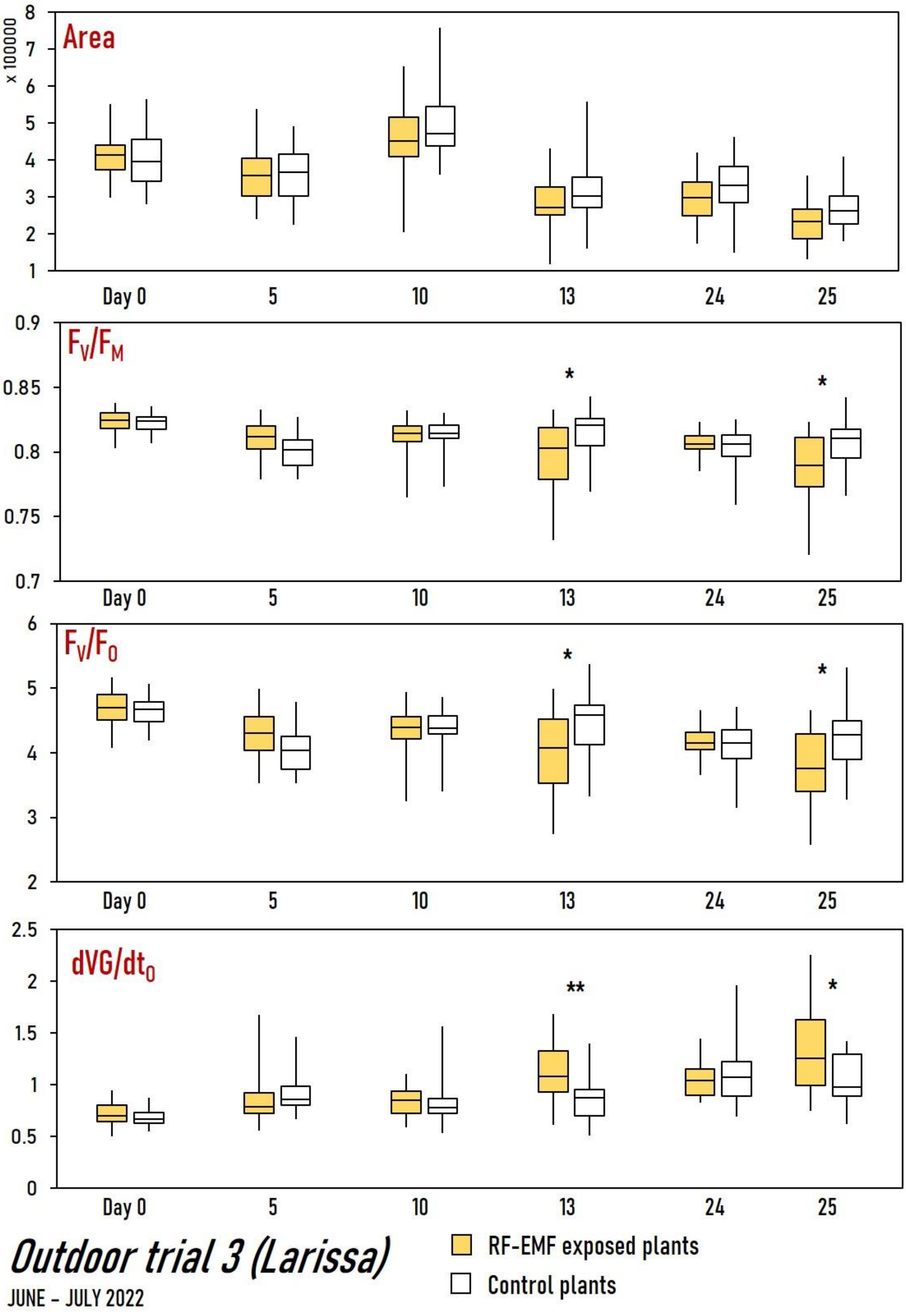

x 100000
Area
Day 0
5
10
13
24
25
F_V/F_M
*
*
F_V/F_0
*
*
dVG/dt_0
**
*
Outdoor trial 3 (Larissa)
JUNE – JULY 2022
RF-EMF exposed plants
Control plants

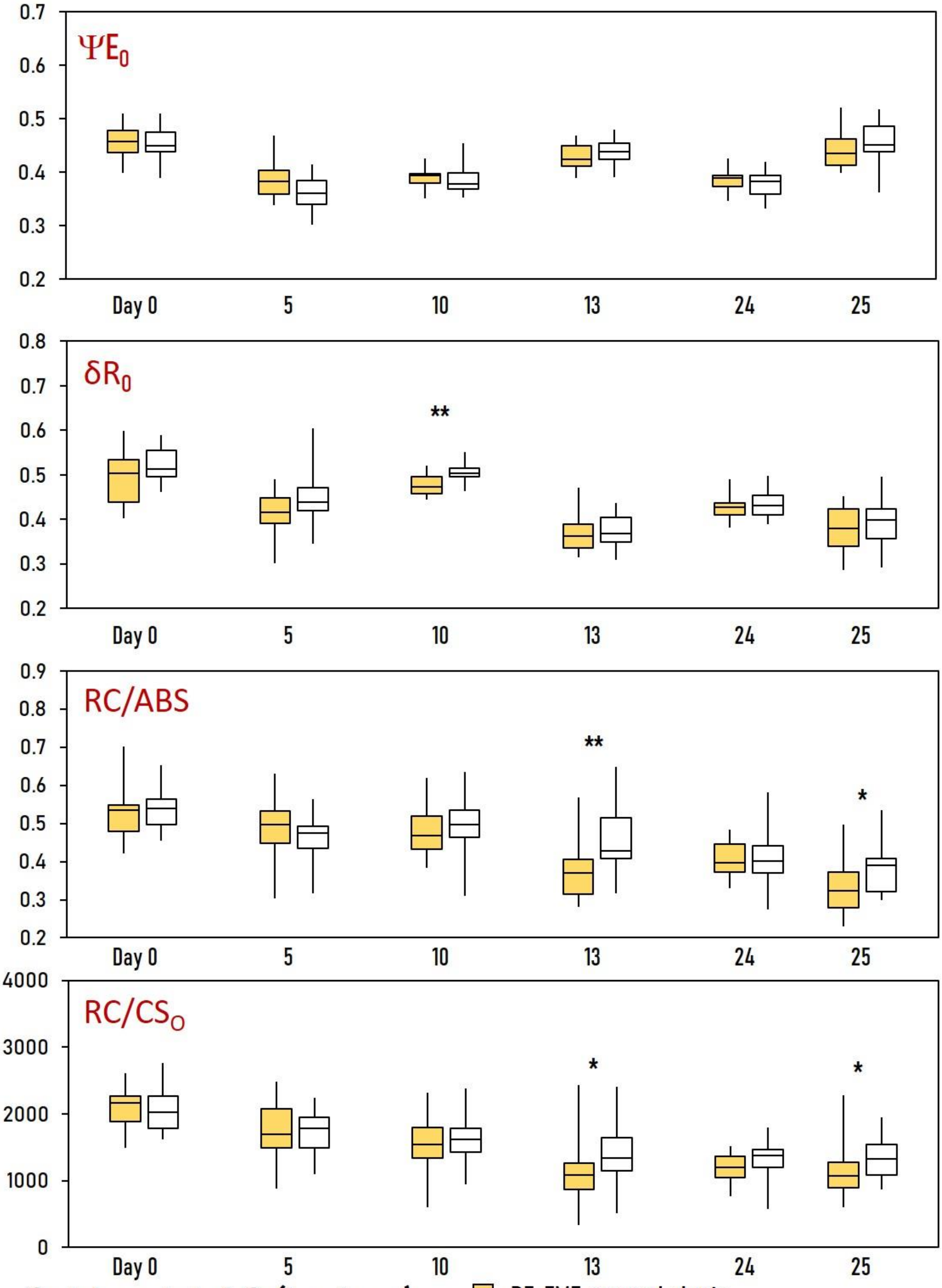

Outdoor trial 3 (Larissa)

JUNE – JULY 2022

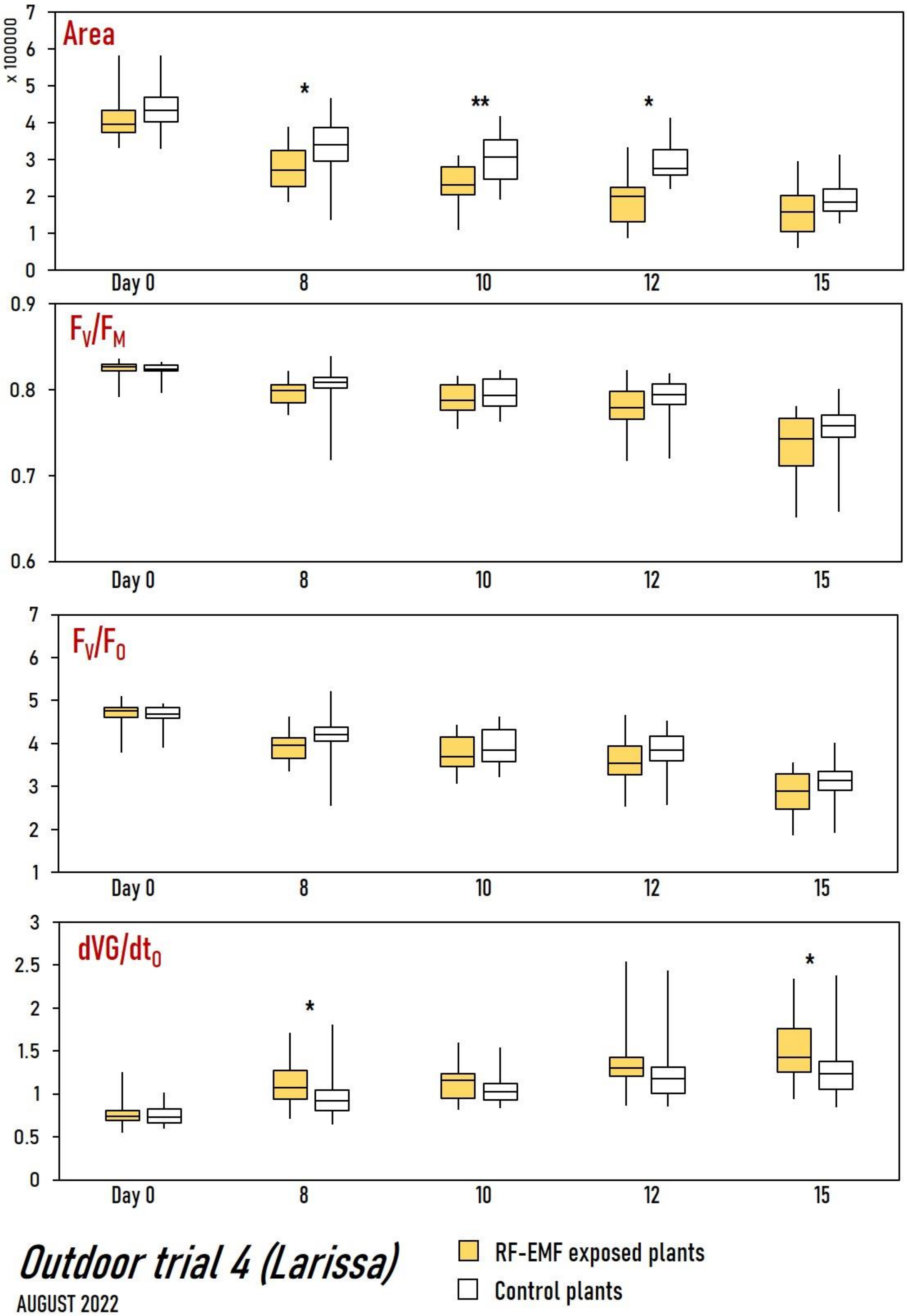

Outdoor trial 4 (Larissa)
AUGUST 2022

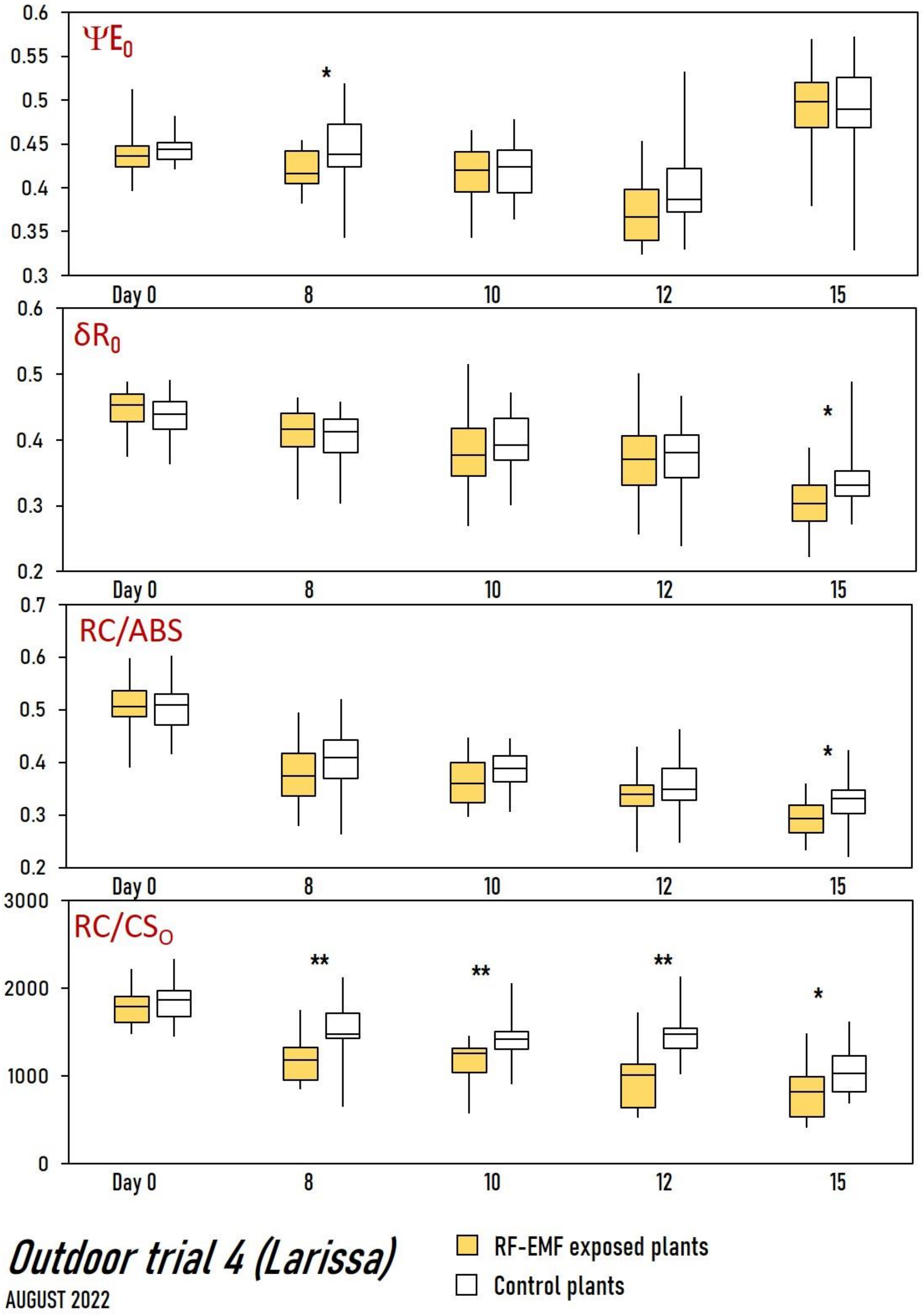
ΨE0
δR0
RC/ABS
RC/CSO
Day 0
8
10
12
15
Outdoor trial 4 (Larissa)
AUGUST 2022
RF-EMF exposed plants
Control plants

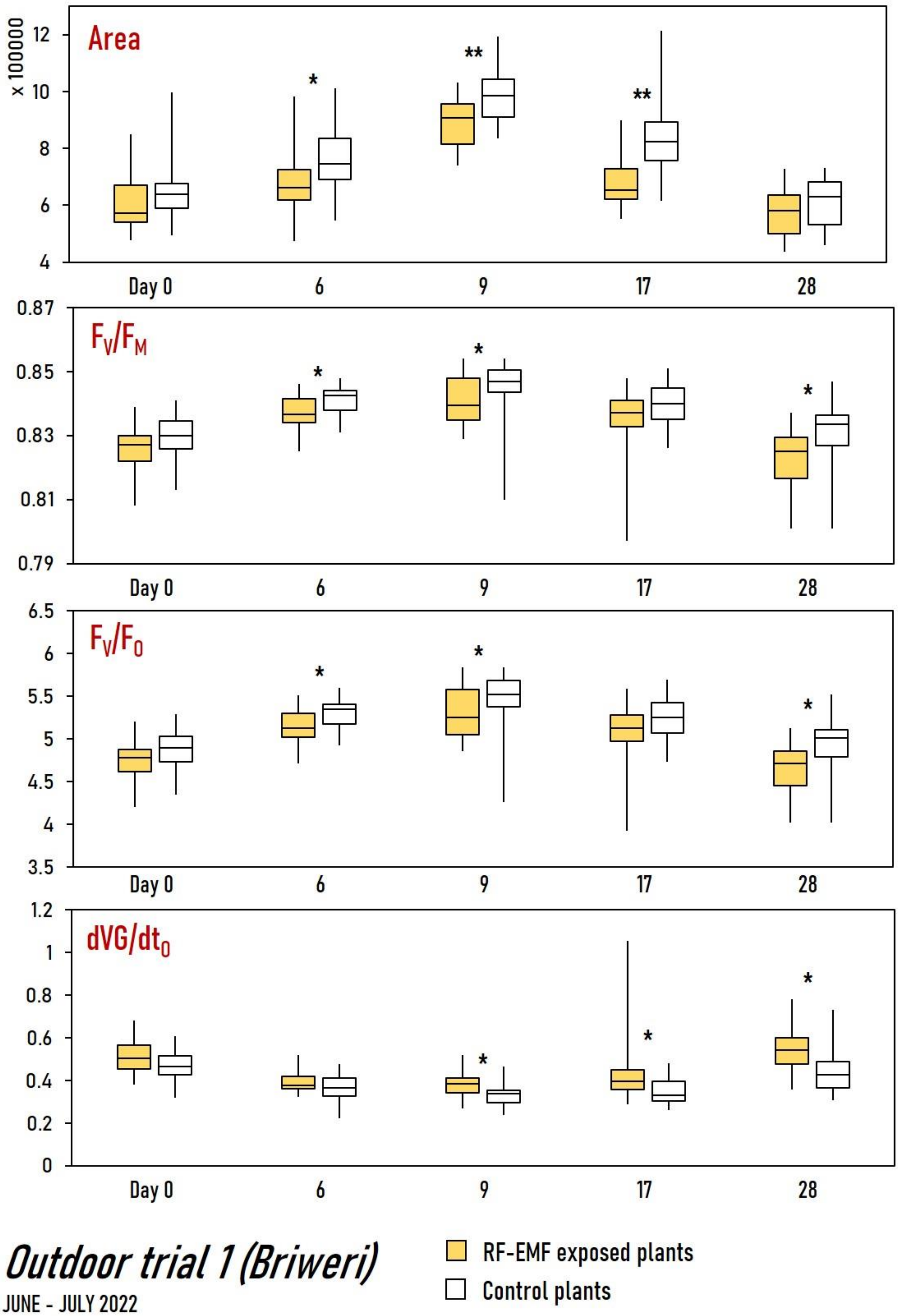

Outdoor trial 1 (Briweri)

JUNE - JULY 2022

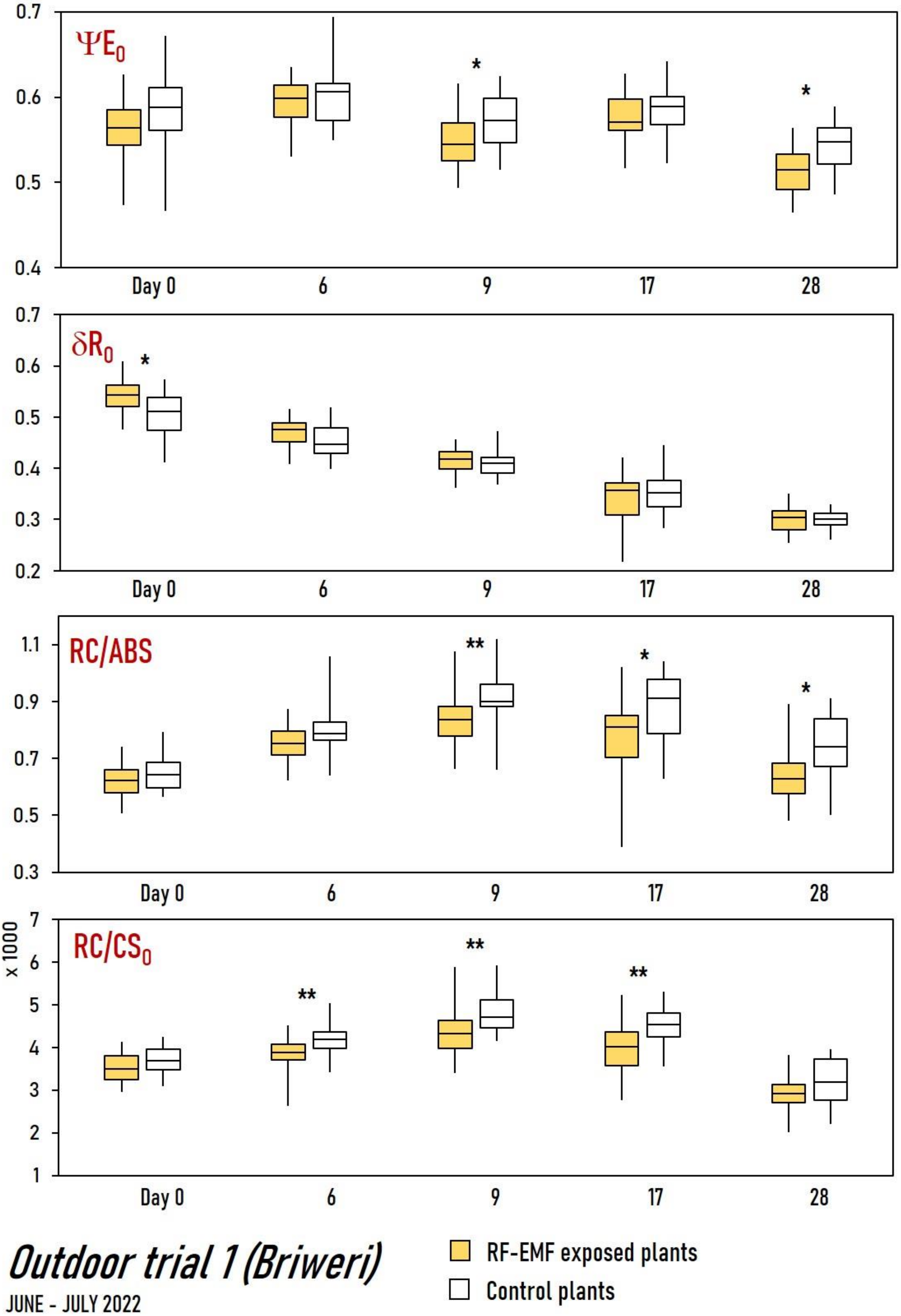

Outdoor trial 1 (Briweri)

JUNE - JULY 2022

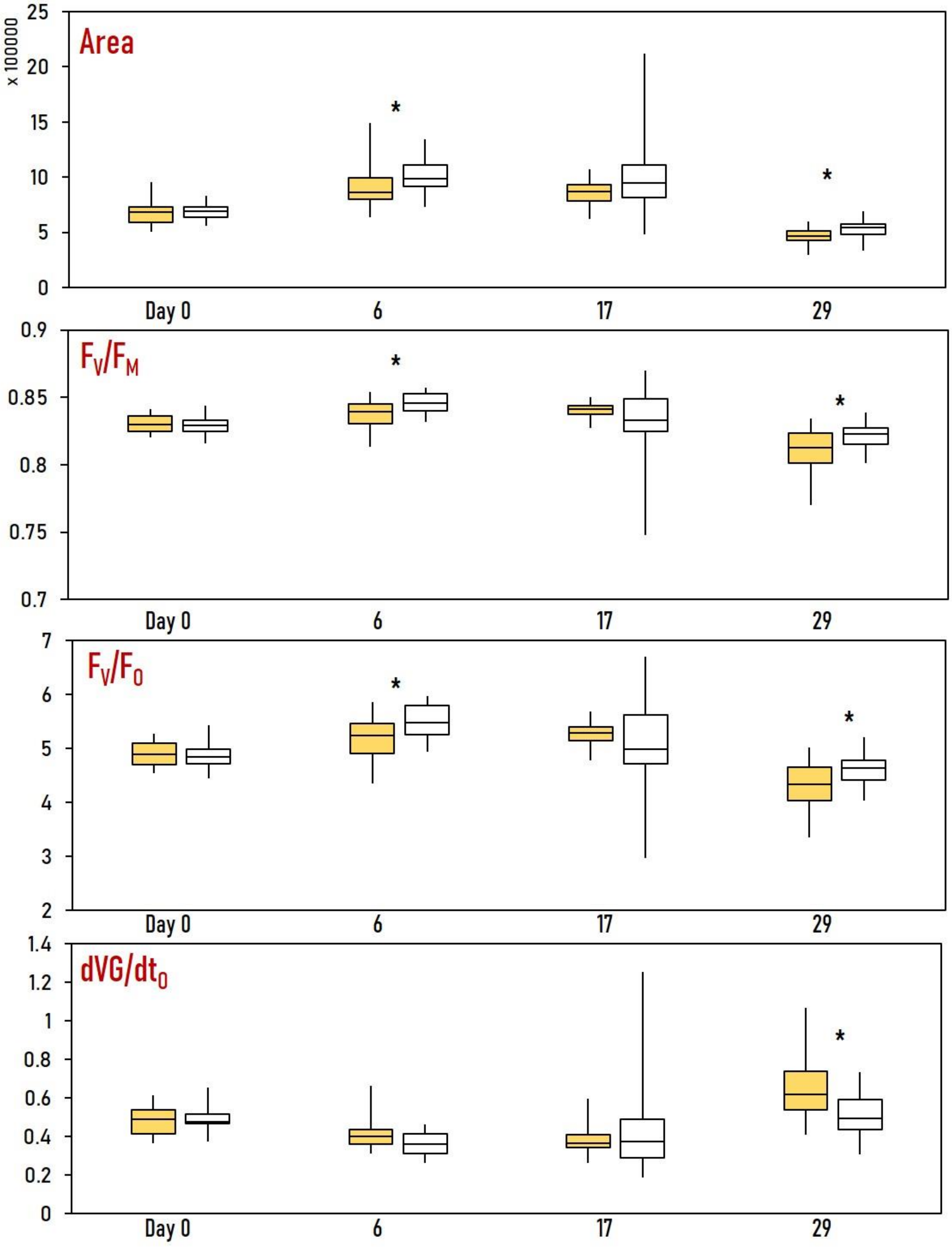

Outdoor trial 2 (Briweri)

JUNE - JULY 2022

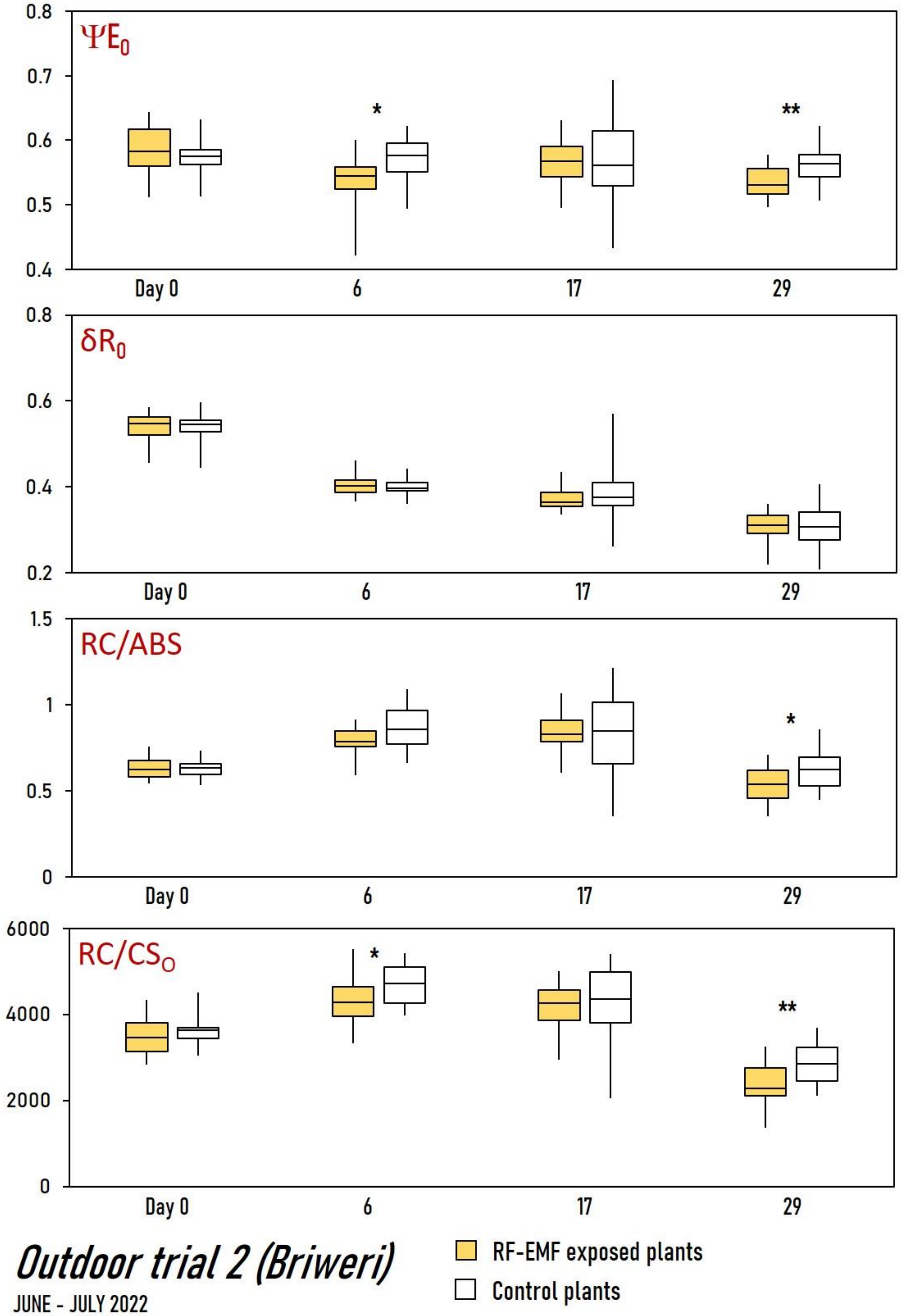

Outdoor trial 2 (Briweri)

JUNE - JULY 2022

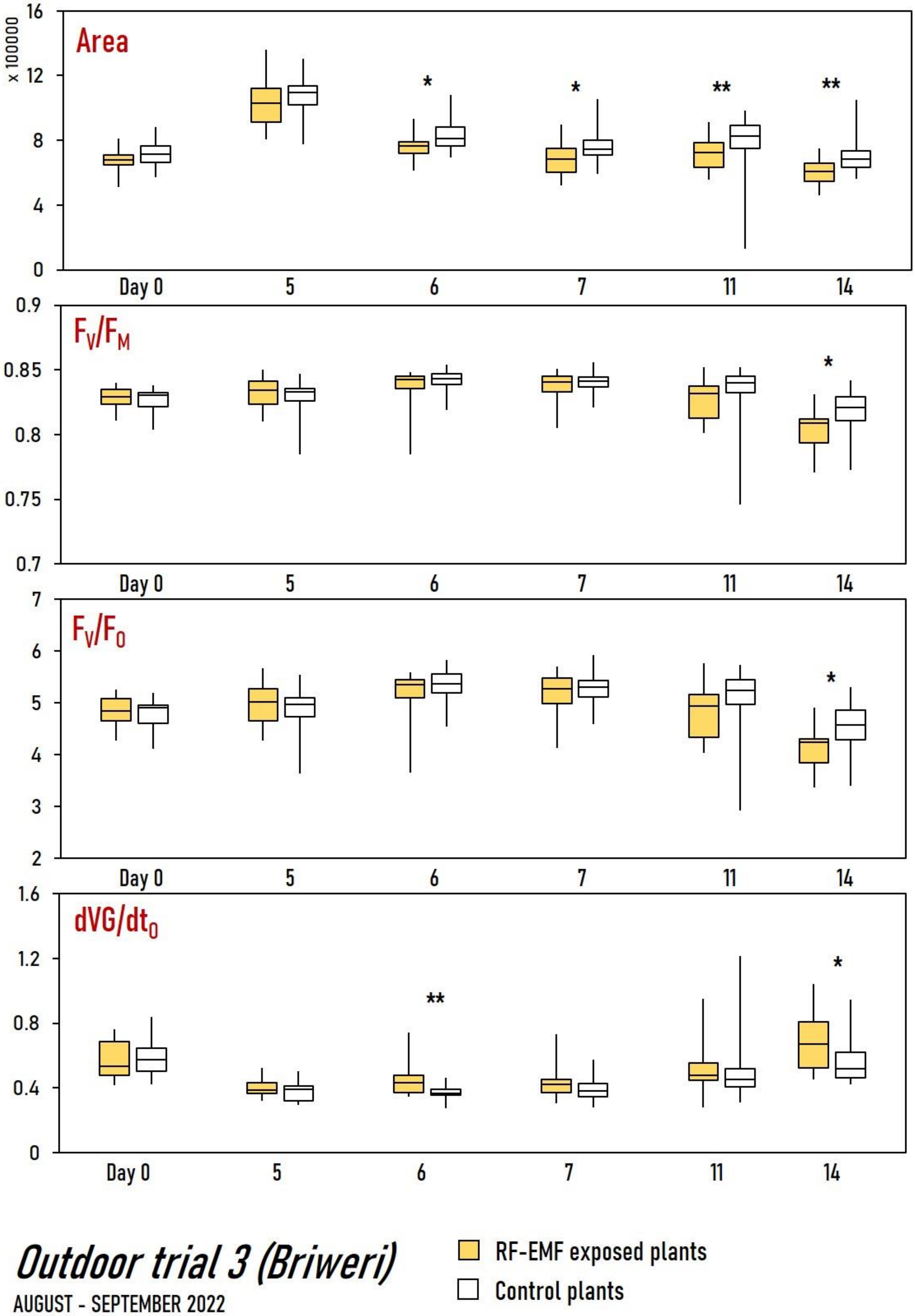

Outdoor trial 3 (Briweri)

AUGUST - SEPTEMBER 2022

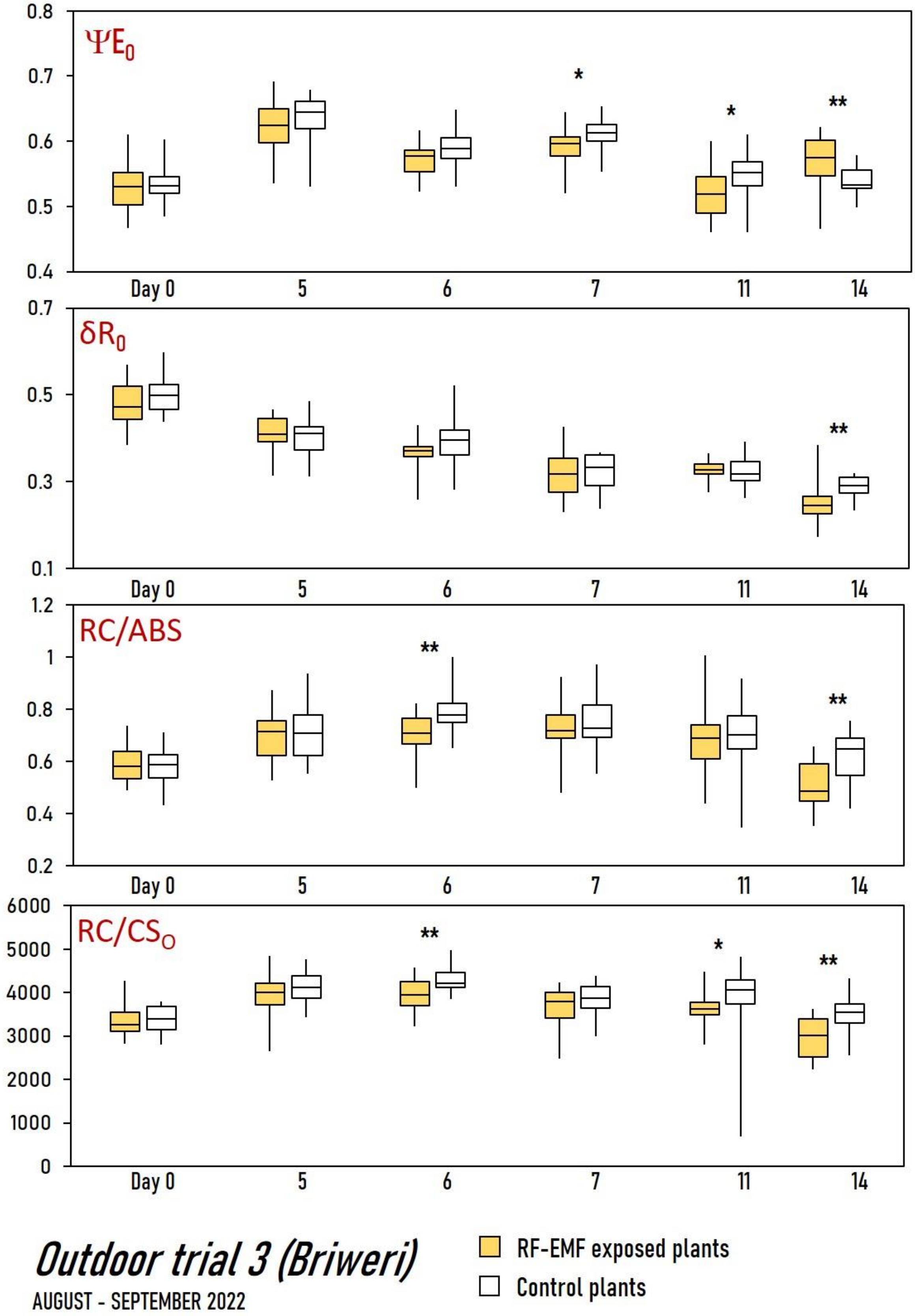
ΨE0
δR0
RC/ABS
RC/CSO
Day 0
5
6
7
11
14
0.8
0.7
0.6
0.5
0.4
0.3
0.1
1.2
1
0.2
6000
5000
4000
3000
2000
1000
0
*
**
Outdoor trial 3 (Briweri)
AUGUST - SEPTEMBER 2022
RF-EMF exposed plants
Control plants

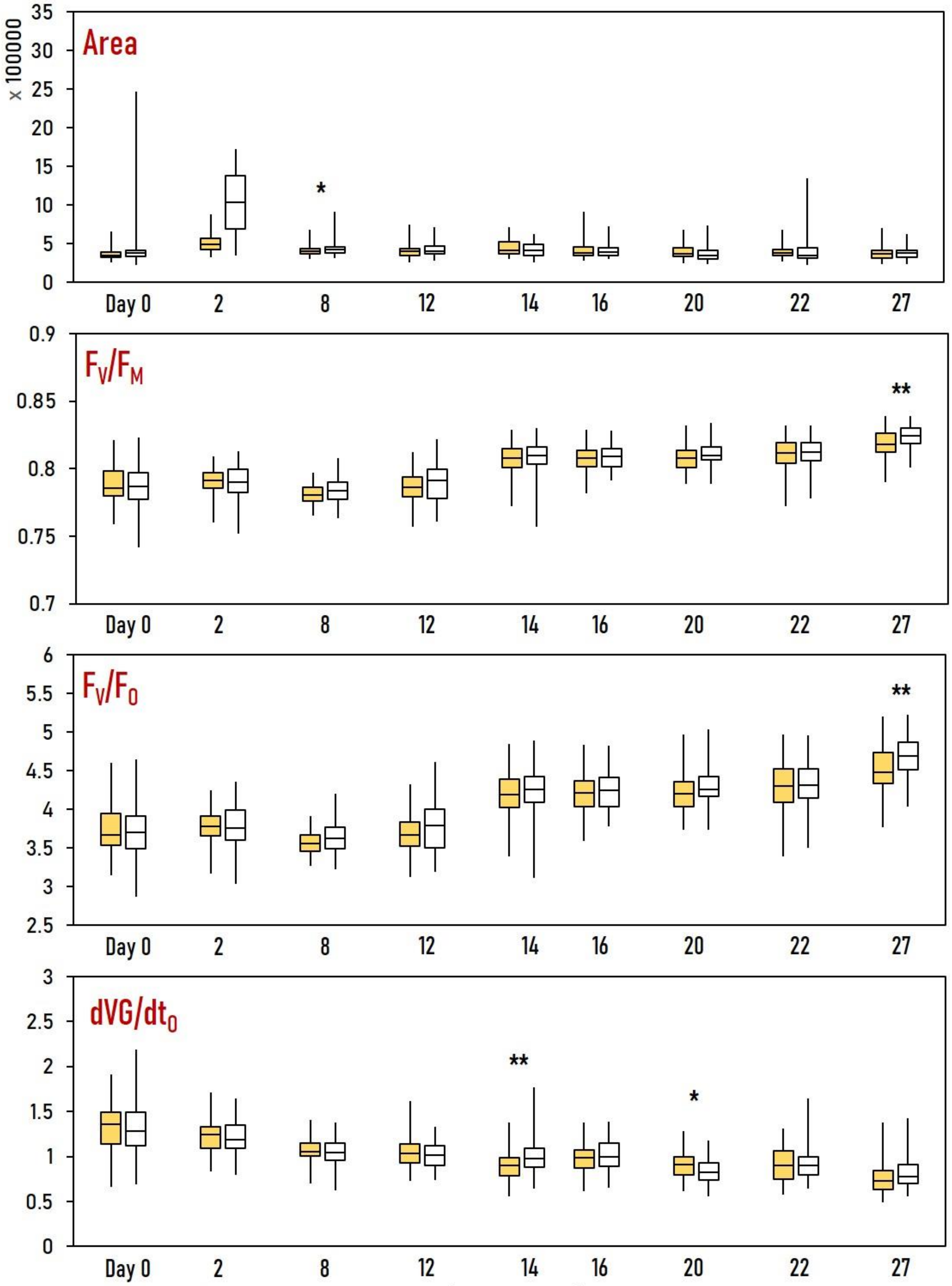

Greenhouse experiment (Larissa)

SEPTEMBER 2022

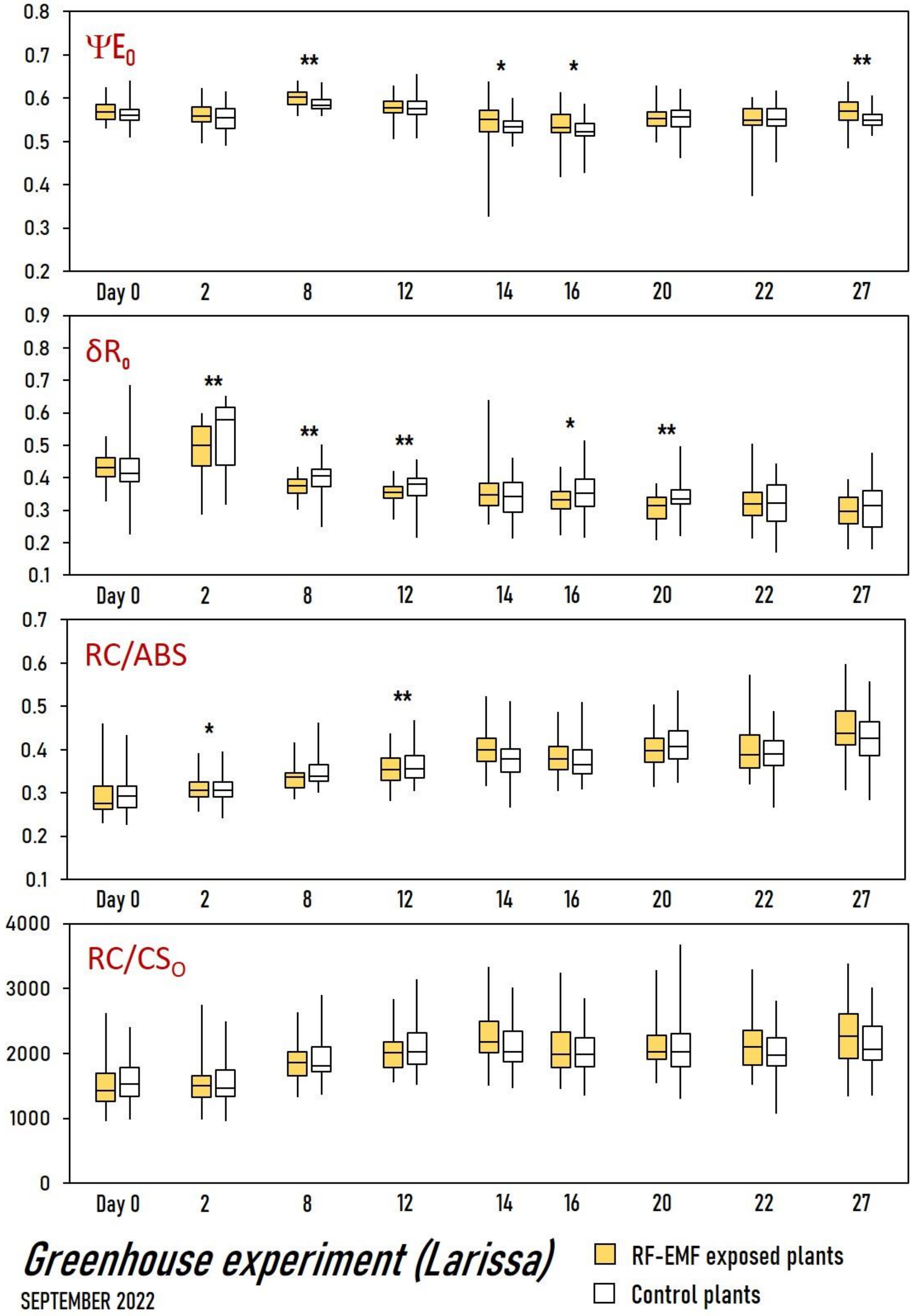
ΨE0
δR0
RC/ABS
RC/CSO
Day 0
2
8
12
14
16
20
22
27
Greenhouse experiment (Larissa)
SEPTEMBER 2022
RF-EMF exposed plants
Control plants

Following diagrams show if the differences between RF-EMF exposed and control plants with relation to the 8 OJIP parameters at each measurement time point were statistically significant difference ($p < 0.05$) and if the values from exposed plants were greater or smaller than from control plants.

Area

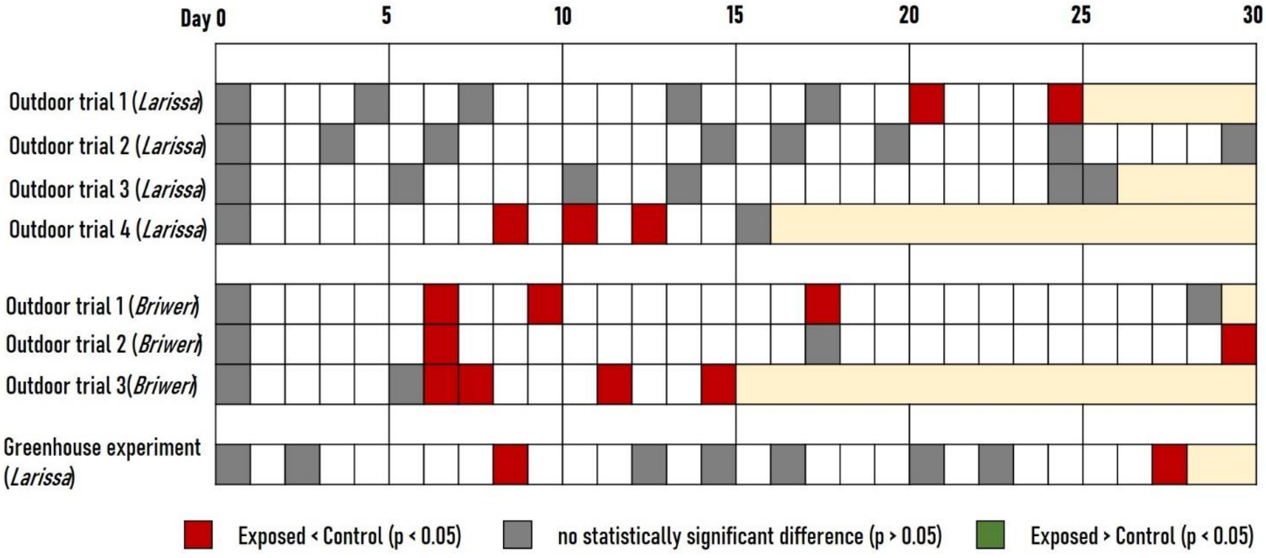

F_V/F_M

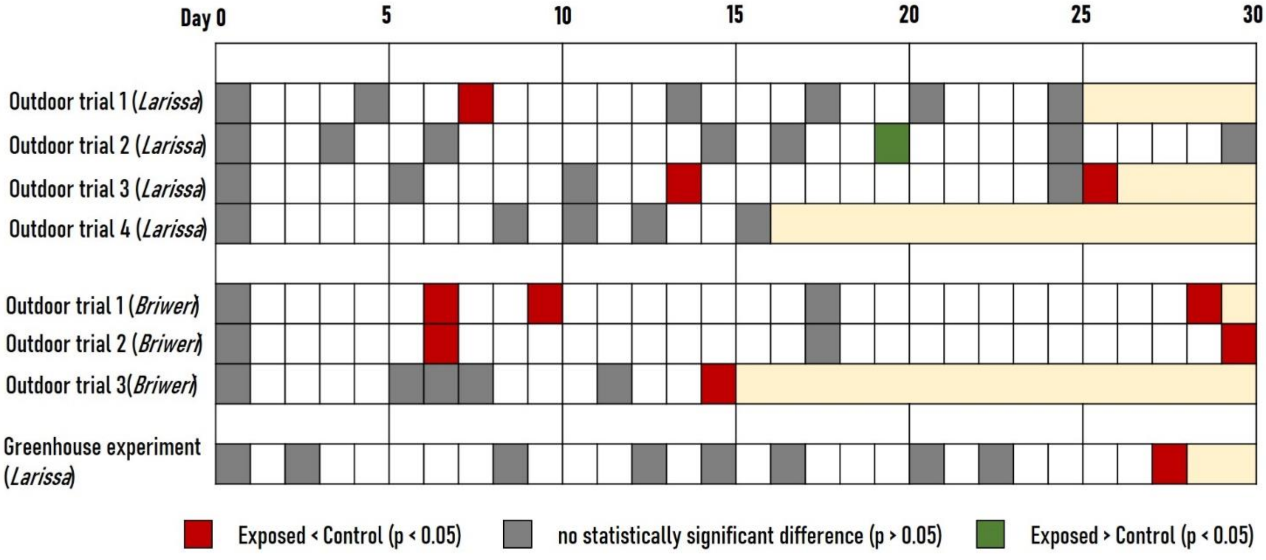

F_V/F_0

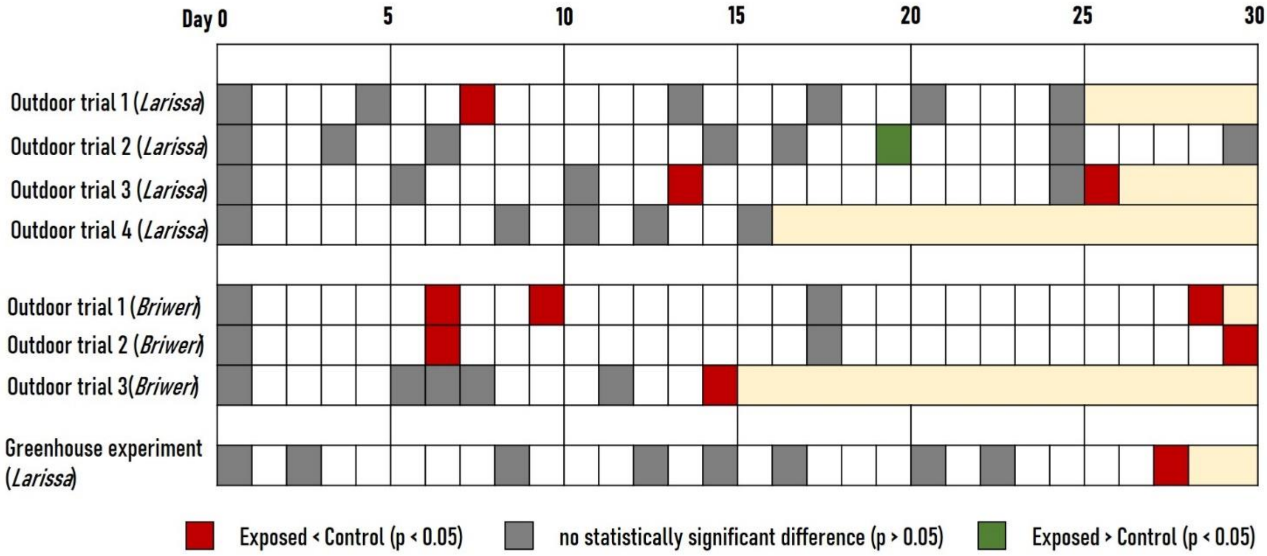
Day 0
5
10
15
20
25
30
Outdoor trial 1 (*Larissa*)
Outdoor trial 2 (*Larissa*)
Outdoor trial 3 (*Larissa*)
Outdoor trial 4 (*Larissa*)
Outdoor trial 1 (*Briweri*)
Outdoor trial 2 (*Briweri*)
Outdoor trial 3(*Briweri*)
Greenhouse experiment (*Larissa*)
Exposed < Control (p < 0.05)
no statistically significant difference (p > 0.05)
Exposed > Control (p < 0.05)

dVG/dt_0

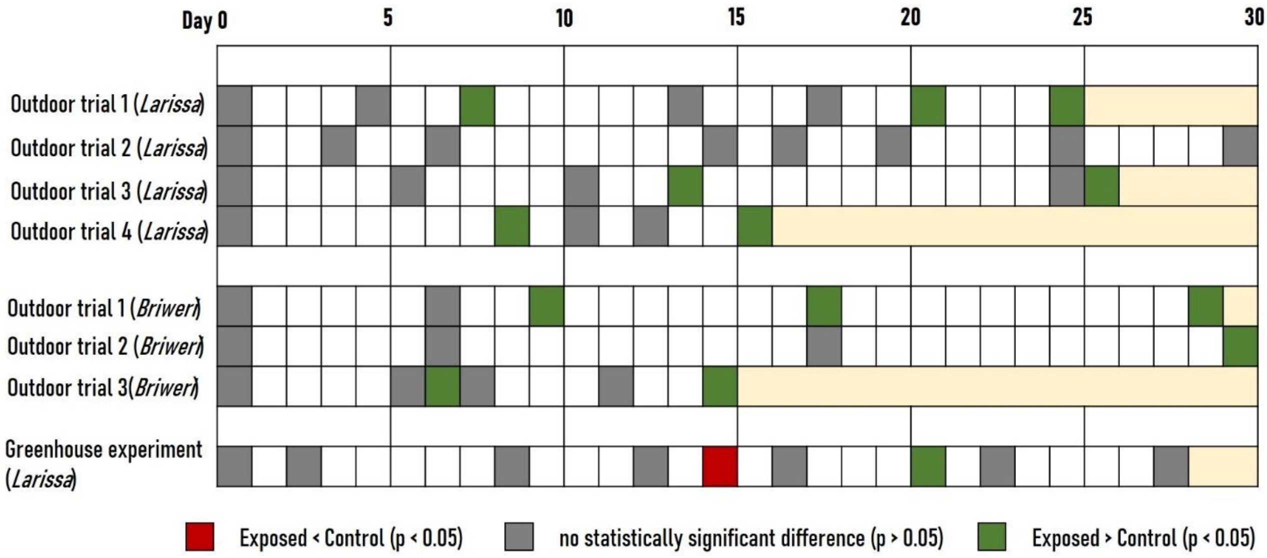
Day 0
5
10
15
20
25
30
Outdoor trial 1 (*Larissa*)
Outdoor trial 2 (*Larissa*)
Outdoor trial 3 (*Larissa*)
Outdoor trial 4 (*Larissa*)
Outdoor trial 1 (*Briweri*)
Outdoor trial 2 (*Briweri*)
Outdoor trial 3(*Briweri*)
Greenhouse experiment (*Larissa*)
Exposed < Control (p < 0.05)
no statistically significant difference (p > 0.05)
Exposed > Control (p < 0.05)

ΨE_0

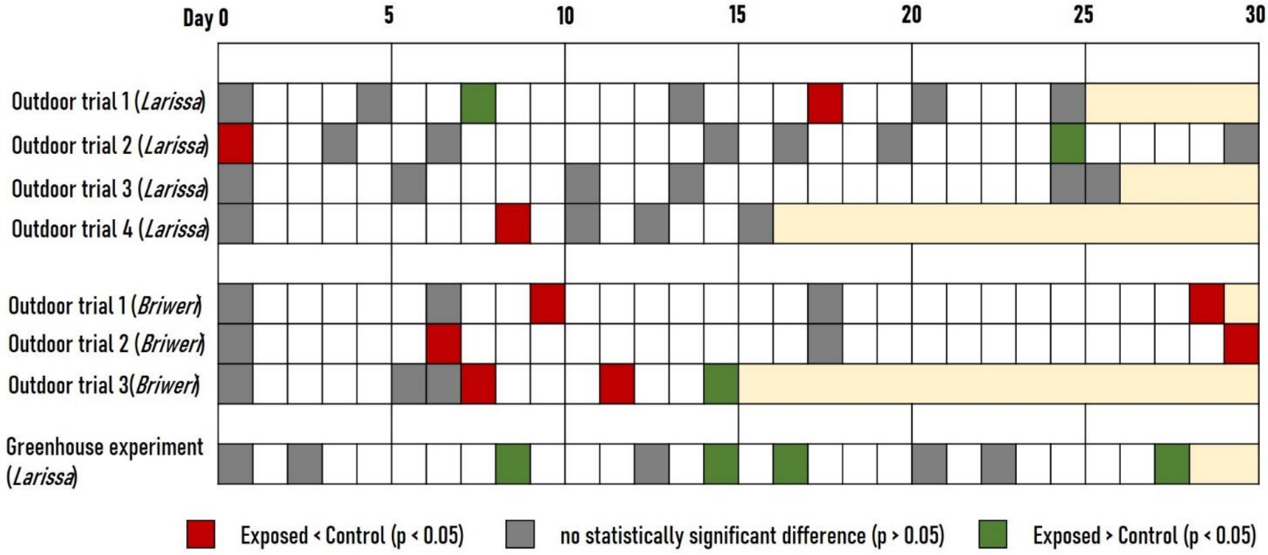

δR_0

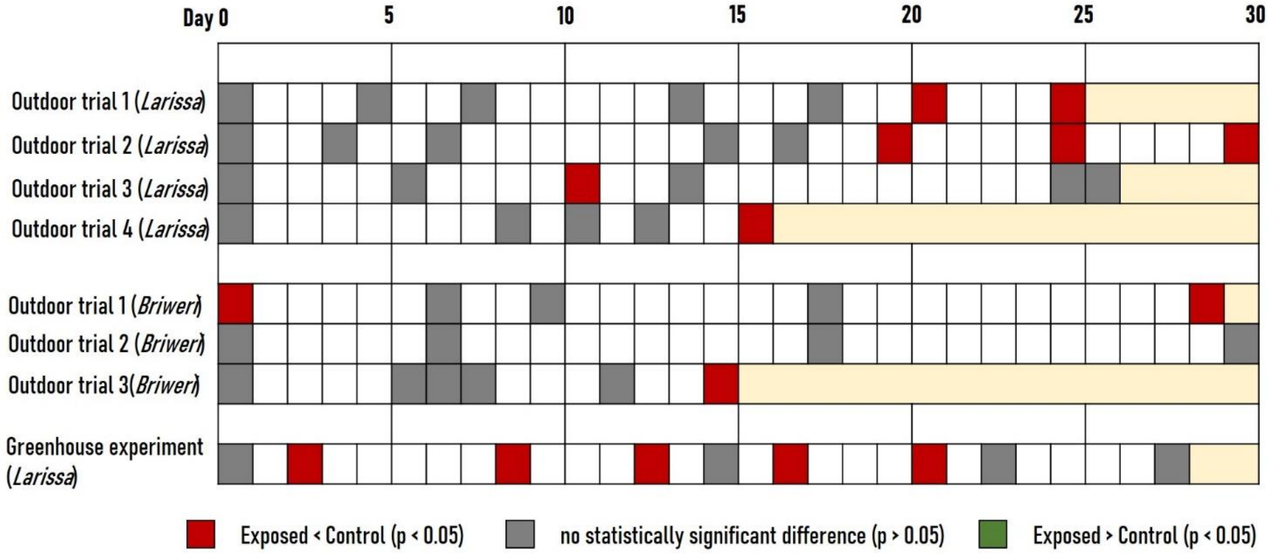

RC/ABS

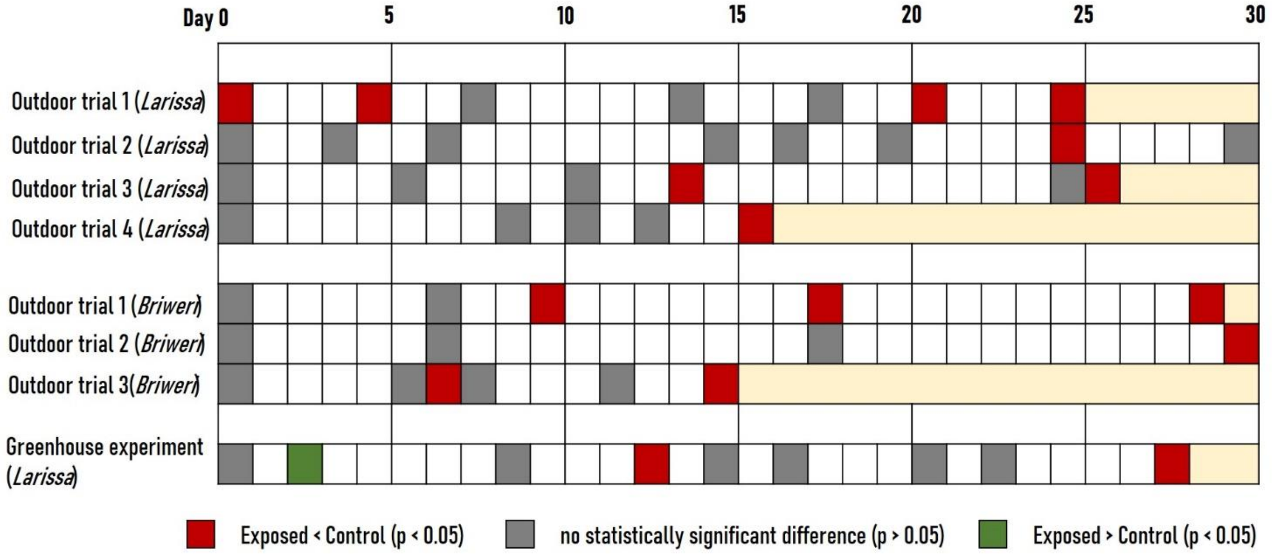

Day 0
5
10
15
20
25
30
Outdoor trial 1 (*Larissa*)
Outdoor trial 2 (*Larissa*)
Outdoor trial 3 (*Larissa*)
Outdoor trial 4 (*Larissa*)
Outdoor trial 1 (*Briweri*)
Outdoor trial 2 (*Briweri*)
Outdoor trial 3(*Briweri*)
Greenhouse experiment (*Larissa*)
Exposed < Control (p < 0.05)
no statistically significant difference (p > 0.05)
Exposed > Control (p < 0.05)

RC/CS$_0$

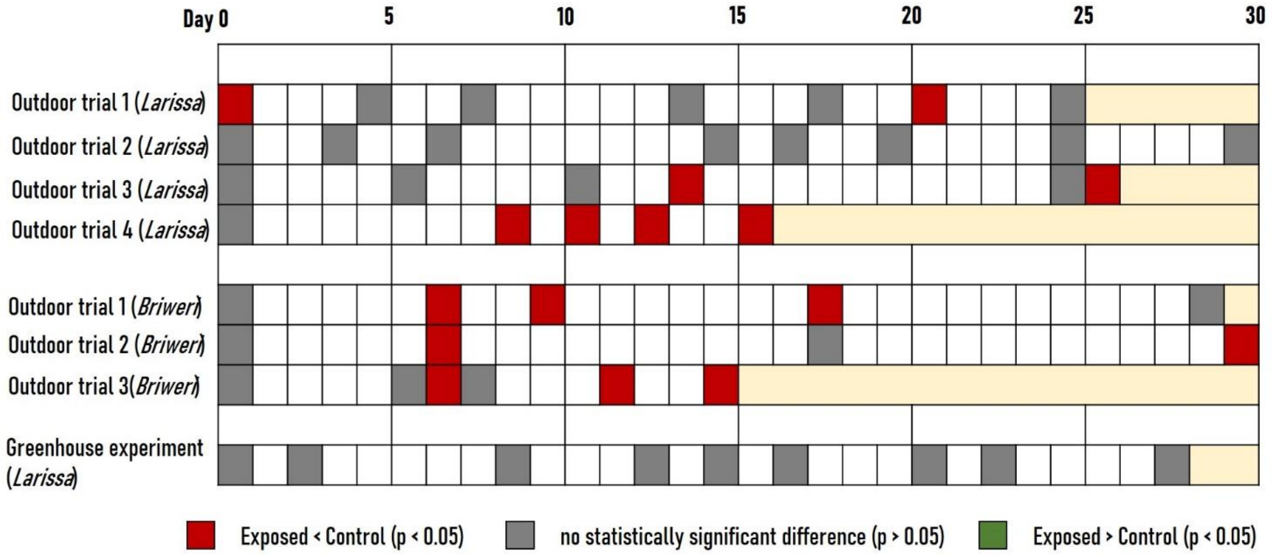

Day 0
5
10
15
20
25
30
Outdoor trial 1 (*Larissa*)
Outdoor trial 2 (*Larissa*)
Outdoor trial 3 (*Larissa*)
Outdoor trial 4 (*Larissa*)
Outdoor trial 1 (*Briweri*)
Outdoor trial 2 (*Briweri*)
Outdoor trial 3(*Briweri*)
Greenhouse experiment (*Larissa*)
Exposed < Control (p < 0.05)
no statistically significant difference (p > 0.05)
Exposed > Control (p < 0.05)

FIELD TRIAL'S METEOROLOGICAL DATA

On the Forschungsring e.V. experimental's field (coordinates 49°49'57.4 "N 8°34'22.2 "E), the following meteorological data were collected: August – September 2021 (Outdoor trial 1 - *Larissa*); October-November 2021 (Outdoor trial 2 - *Larissa*); June- July 2022 (Outdoor trial 3 – *Larissa*, Outdoor trials 1,2 – *Briweri*); August 2022 (Outdoor trial 4 – *Larissa*, Outdoor trials 3 – *Briweri*).

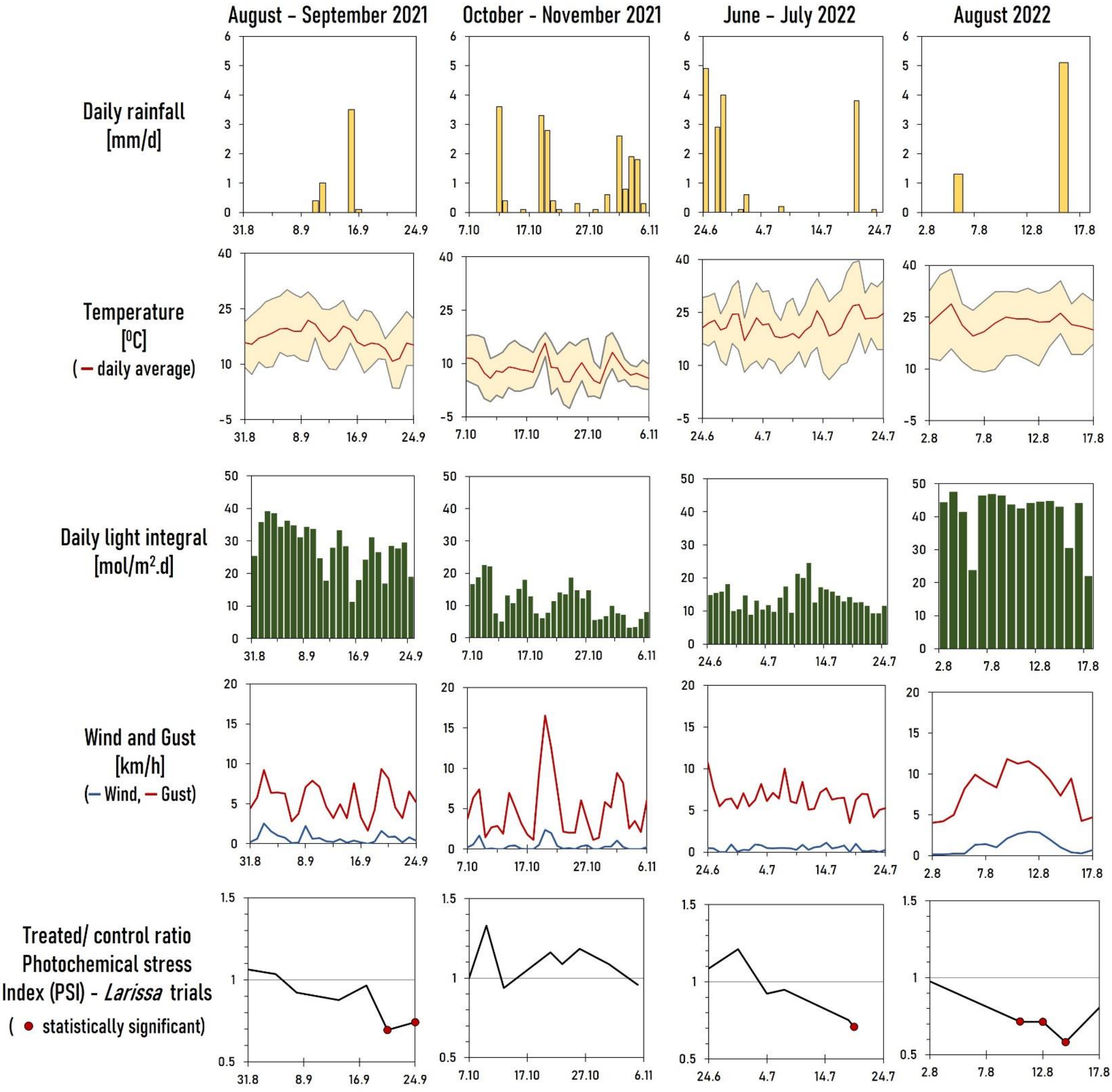

Supplement: Supplementary file 1 [file plants-12-01082-s001.zip › plants-2211548-supplementary.pdf]
